# Long-Term Warming Alters Carbohydrate Degradation Potential in Temperate Forest Soils

**DOI:** 10.1128/AEM.02012-16

**Published:** 2016-10-27

**Authors:** Grace Pold, Andrew F. Billings, Jeff L. Blanchard, Daniel B. Burkhardt, Serita D. Frey, Jerry M. Melillo, Julia Schnabel, Linda T. A. van Diepen, Kristen M. DeAngelis

**Affiliations:** a Graduate Program in Organismic and Evolutionary Biology, University of Massachusetts, Amherst, Massachusetts, USA; b Department of Microbiology, University of Massachusetts, Amherst, Massachusetts, USA; c Department of Biology, University of Massachusetts, Amherst, Massachusetts, USA; d Department of Natural Resources and the Environment, University of New Hampshire, Durham, New Hampshire, USA; e The Ecosystems Center, Marine Biological Laboratories, Woods Hole, Massachusetts, USA; University of Tennessee and Oak Ridge National Laboratory

## Abstract

As Earth's climate warms, soil carbon pools and the microbial communities that process them may change, altering the way in which carbon is recycled in soil. In this study, we used a combination of metagenomics and bacterial cultivation to evaluate the hypothesis that experimentally raising soil temperatures by 5°C for 5, 8, or 20 years increased the potential for temperate forest soil microbial communities to degrade carbohydrates. Warming decreased the proportion of carbohydrate-degrading genes in the organic horizon derived from eukaryotes and increased the fraction of genes in the mineral soil associated with Actinobacteria in all studies. Genes associated with carbohydrate degradation increased in the organic horizon after 5 years of warming but had decreased in the organic horizon after warming the soil continuously for 20 years. However, a greater proportion of the 295 bacteria from 6 phyla (10 classes, 14 orders, and 34 families) isolated from heated plots in the 20-year experiment were able to depolymerize cellulose and xylan than bacterial isolates from control soils. Together, these findings indicate that the enrichment of bacteria capable of degrading carbohydrates could be important for accelerated carbon cycling in a warmer world.

**IMPORTANCE** The massive carbon stocks currently held in soils have been built up over millennia, and while numerous lines of evidence indicate that climate change will accelerate the processing of this carbon, it is unclear whether the genetic repertoire of the microbes responsible for this elevated activity will also change. In this study, we showed that bacteria isolated from plots subject to 20 years of 5°C of warming were more likely to depolymerize the plant polymers xylan and cellulose, but that carbohydrate degradation capacity is not uniformly enriched by warming treatment in the metagenomes of soil microbial communities. This study illustrates the utility of combining culture-dependent and culture-independent surveys of microbial communities to improve our understanding of the role changing microbial communities may play in soil carbon cycling under climate change.

## INTRODUCTION

Microbes are central players in the soil carbon (C) cycle, processing litter and soil carbon to biomass and CO_2_ as a function of the quantity and quality of carbon available, genetically encoded potential for targeting that carbon, and temperature. Global climate warming is expected to alter microbial activity in soils by directly accelerating enzyme process rates (1–3), and potentially indirectly through its impacts on soil microbial community composition. Numerous studies have examined how climate warming has affected soil microbial communities (4), but it is difficult to draw conclusions about shifts in the functional capacities of communities based on the phylogenetic marker genes used in these studies, because many traits involved in carbon cycling in bacteria are only shallowly phylogenetically conserved (5). Therefore, a direct assessment of pertinent genetic and physiological functions in microbial communities subject to warming is necessary to infer potential linkages between changes in microbial community structure and changes in community carbon cycling activity.

As the first and often rate-limiting step of decomposition (6), microbes must depolymerize compounds, such as the abundant carbohydrates hemicellulose and chitin, before the resultant monomers can be taken up and used for metabolism. This requires the production and release of enzymes into the soil, where the optimum temperature for activity, rate of substrate turnover, and rate of activity for a given substrate concentration differ between the “isoenzymes” produced by different members of the microbial community (7). These enzymes tend to depolymerize their substrates faster at higher temperatures (2, 8), but soil-warming-induced shifts in both the soil carbon pool and isoenzymes produced by the microbial community may also influence carbon cycling activity (9). In both tundra (10, 11) and temperate grassland ecosystems (9, 12), increases in the size of substrate pools have accompanied generally small (∼5 to 10%) but significant increases in the genes encoding the enzymes responsible for the degradation and uptake of carbohydrates, such as starch and cellulose. However, because of temporal mismatches in microbial and dominant vegetation response to warming, potential carbohydrate decomposition of soil microbial communities in tundra or grassland ecosystems may respond to climate warming very differently than those of forests.

One such factor that may affect how forests respond to climate warming is the strong vertical stratification of soil physical structure, which was not considered in previous mineral-only prairie soils (12) or peat-only tundra soils (10, 11). Forest soil profiles typically consist of a root-entangled mat of partially decomposed plant litter (organic horizon) that sits atop a less-carbon-rich mineral soil. The functional (13) and taxonomic (14) compositions of microbial communities are similarly stratified, with the organic horizon more enriched in fungi and carbohydrate-degrading genes than the mineral soil (15). Forest organic horizon communities have previously been shown to respond to environmental stressors with greater functional (15) and taxonomic (16) shifts than those of the mineral soil, indicating that evaluating the horizon-specific responses to warming may provide insight into altered microbial carbon cycling capacity.

To determine how climate change may affect carbon cycling in temperate forest ecosystems over long time periods, three chronic soil-warming experiments were established in central Massachusetts five (17), eight (18), and 20 years prior to sample collection for the present study (19) (Table 1). In all experiments, carbon mineralization increased with the onset of warming (17–19), in line with expectations based on knowledge of the response of basic enzyme kinetics to elevated temperatures (8). However, respiration has not remained consistently higher in warmed than in control plots at the longest-running site and declined to a rate similar to that of the control treatment after approximately a decade of warming (20). This coincided with a depletion of readily available soil carbon in heated plots (21), a decrease in microbial biomass, a shift in the microbial community toward Gram-positive bacteria (22), and acclimation of respiration to a lower-than-expected rate as temperature increased (21). However, by the time soil samples were collected for the present study, respiration was again higher in the heated plots than in the control plots (23). This made a third phase in the respiration response to warming and was associated with substantial shifts in the physiology (24) and identity (4, 16) of microbial communities. In contrast, no such shifts in the bacterial community were seen at the time of sampling in the other two experiments (16), although substantial shifts in the quality of soil carbon available to microbes have been observed (25). Whether shifts in the microbial community responsible for the rate-limiting depolymerization step of decomposition have also occurred at the 20-year site but not the 5- and 8-year warming sites is an open question.

**TABLE 1:** Summary of warming experiments used in this study

Characteristic^*a*^	SWaN plots (17)	Barre Woods (18)	Prospect Hill (19)
Latitude, longitude	42.54°N, 72.18°W	42°28′N, 72°10′W	42.54°N, 72.18°W
Yr started (duration [yr] at time of soil collection for metagenome)	2006 (5)	2003 (8)	1991 (20)
Plot size (m)	3 by 3	30 by 30	6 by 6
No. of plots	6	1 megaplot with 25 subplots	6
Soil pH, O-horizon	3.72	4.29	3.82
Soil pH, 0- to 10-cm mineral	4.38	4.42	4.41
Total C (mean ± SE) (g of C · m^−2^)			
O-horizon	3,314 (404)	1,772 (621)	2,565 (247)
0- to 10-cm mineral	3,478 (121)	1,810 (92)	2,859 (444)
Moisture (control, warmed) (g of H_2_O · g^−1^ of soil [dry wt])			
O-horizon	1.59, 1.26	1.32, 1.02	1.49, 0.99
0- to 10-cm mineral	0.41, 0.40	0.37, 0.31	0.44, 0.38
Dominant overstory vegetation	Acer rubrum, Acer pensylvanicum, Betula papyrifera, Fagus grandifolia, Quercus rubra, Quercus velutina	A. rubrum, Fraxinus americana, Q. rubra, Q. velutina	A. rubrum, A. pensylvanicum, B. papyrifera, Q. velutina
Soil series	Gloucester	Canton	Gloucester

a Soil pH and carbon data refer to control plots only. O-horizon, organic horizon.

Both culture-dependent and culture-independent approaches were used here to evaluate how chronic warming affects the microbial communities responsible for carbohydrate depolymerization in temperate forest soils. We used metagenomics to determine whether soil warming has preferentially enriched the ability to depolymerize carbohydrates in the organic horizon after 20 years of warming, or whether this effect is also visible in the 5- and 8-year experiments. This approach has the strength of informing our understanding of how chronic warming changes the functional potential of whole communities (12). We also evaluated the hypothesis that 20 years of chronic warming has increased the capacity of bacteria isolated from experimentally heated plots to depolymerize cellulose, chitin, and xylan compared to their control plot counterparts when assayed in the laboratory, as would be expected if warming had promoted the expression of these traits in organisms.

## MATERIALS AND METHODS

### Experimental site.

Soils were studied from three soil-warming experiments of various durations at the Harvard Forest Long Term Ecological Research (LTER) site in Petersham, MA (Table 1). As in other metagenomic studies (15), we used space-for-time substitution to infer how the effect of warming treatment on microbial functional potential may vary over time. Two of the experiments, Prospect Hill and SWaN (Soil Warming and Nitrogen) are located immediately adjacent to one another in the main tract of the forest and had been running for 20 years and 5 years, respectively, at the time of soil collection for metagenomic analysis. The third experiment, Barre Woods, is located approximately 5 km east of the main tract and had been running for 8 years when soils were collected for metagenomic analysis. Resistance cables buried at a 10-cm depth in the soil maintain soil temperatures in heated plots consistently 5°C above the temperatures observed in the control plots, as regulated by a data logger that calculates the mean temperatures in warmed and control plots each 10 min from thermistors buried in each plot and switches the warming cables on or off accordingly (19). All experiments are located in mixed deciduous forest stands (Table 1). An easily distinguished organic mat overlies a deep well-drained mineral soil, which is of the Gloucester series at Prospect Hill and SWaN (19) and of the Canton series at Barre Woods (18). Precipitation is distributed approximately evenly throughout the year, with an average of 118 cm per year since 1991. Mean monthly temperatures range from −6°C in January to 20°C in July (94). Soil temperatures at the 10-cm depth averaged 9 to 11°C in the control plots on the days samples were collected.

### Soil collection.

On 25 to 27 October 2011, organic horizon material was collected as intact 20- by 20-cm blocks, while mineral horizon soil (0 to 10 cm) was collected directly under each organic horizon sample using a 9-cm-diameter custom stainless steel auger. Organic horizon and mineral soil samples were collected from four plots in each experiment, for a total of 48 samples (3 experiments × 2 warming treatments × 2 soil horizons × 4 replicates). In the field, subsamples from both horizons were immediately flash-frozen and stored at −80°C until nucleic acid extraction.

### Nucleic acid extraction.

Complete nucleic acids were extracted from soils using a modified cetyltrimethylammonium bromide (CTAB) procedure, as per DeAngelis et al. (16). This method has two bead-beating steps, which are expected to reduce extraction bias against fungi (26). The three extractions were then pooled, and DNA and RNA were separated using an AllPrep DNA/RNA kit (Qiagen). DNA was quantified using the Quant-iT PicoGreen double-stranded DNA (dsDNA) assay kit (Invitrogen), according to the product instructions.

Shotgun metagenome library preparation, sequencing, assembly, and annotation were completed at the Joint Genome Institute using standard operating procedures and pipelines. Unamplified libraries were prepared for each sample using a modified version of the Illumina TruSeq protocol with 500 ng of purified soil DNA. DNA was mechanically sheared to a median length of 270 bp using a Covaris LE220 and then size-selected using solid-phase reversible immobilization. Fragments were then end repaired, and poly(A) tails were added prior to ligation to barcoded sequencing adaptors. After the quantification of individual libraries using quantitative PCR, 11 to 12 libraries were pooled for sequencing on an Illumina HiSeq 2000 (2 × 150-bp strategy). This resulted in an average ± standard error of the mean (SEM) of 1.387 ± 0.06333 Gb per soil sample (metagenome).

### Data quality control and preliminary assembly.

A sliding window approach with a k-mer length of 28 and a step size of 1 was used to initially trim sequences to exclude bases with quality scores lower than 3, strings of 3+ Ns, and reads with a mean quality score lower than 20. Sequences shorter than 50 bp were removed. This resulted in an average of 27,059,049 quality reads per metagenome. The Joint Genome Institute (JGI) assembled merged paired-end reads further using SOAP*denovo* (27), with default parameters (“-K 81 -p 32 -R -d 1”) and 6 k-mers between 85 and 105 nucleotides (nt). Each contig was subsequently dereplicated, keeping only one copy of a read when the first 5 bp are identical and the reads are >95% identical over their entire length. Contigs shorter than 1,800 bp were assembled using Newbler (Life Technologies, Carlsbad, CA; flags: -tr, -rip, -mi 98, -ml 80) and subsequently combined with those that were longer using minimus2 (flags: -D MINID = 98 -D OVERLAP = 80) (28). The data were then uploaded to Integrated Microbial Genomes (IMG/M) as those that mapped to the assembly (assembled) and those that did not (unassembled) for gene prediction and functional annotation (29). Due to poor initial assembly (median, 0.15% of reads assembled; range, 0.02 to 1.27% of reads), both reads that assembled and those that did not were pooled for the analysis, weighting annotations on the assembled contigs by the mean read depth (30, 31).

Sequencing produced an average ± SEM of 1,387,259,102 ± 63,331,106 bp per metagenome, with a mean fragment length of 270 bp and G+C content of 61.6% ± 0.2%. After quality control and assembly, the mean number of scaffolds ± SEM was 7,012,272 ± 287,725, with 32.3% ± 0.3% genes with predicted protein products and 0.24% ± 0.0028% reads predicted as RNA genes. Overall, there were an average ± SEM of 6.99 × 10^6^ ± 0.28 × 10^6^ (see Data Set S1 in the supplemental material) quality-checked merged paired-end reads per sample.

### Annotation.

Metagenome reads were annotated by the JGI's Integrated Microbial Genomes (IMG) pipeline (32). We assigned taxonomy categories to rRNA reads against the SILVAv119 long subunit (LSU) and short subunit (SSU) databases using the RDP algorithm (33, 34). In the IMG analysis pipeline, the taxonomy of protein-coding genes is determined by clustering reads against the set of all nonredundant sequences for genomes in IMG plus KEGG using usearch (35), with -maxhits = 50 and -minlen = 20. Only the best hit based on identity is kept for taxonomy. We subsequently filtered this set to include only hits with >60% identity at the phylum level and merged it with the Pfam annotations for each read for function × taxonomy analysis. Protein family (Pfam) annotations were made using an HMMER version 3.0 (36) search against the Pfam-A database (37) and filtered to include only those matches with a maximum E value of −5 (38) and a minimum alignment length of 20 amino acids (39). These cutoffs left an average of 41.2% predicted coding sequences (2.94 million reads) annotated to Pfams (see Data Set S1 in the supplemental material).

Pfam annotations were subsequently mapped to carbohydrate-active enzymes (CAZymes) (40) using the R package Pfam.db (version 3.1.2 [https://bioconductor.org/packages/release/data/annotation/html/PFAM.db.html]). We elected to map from Pfams to CAZymes rather than directly to CAZymes so we could (i) simultaneously assess the relative abundance of taxa over all functions and of CAZymes as a fraction of all annotated functions and (ii) reduce the risk of false-positive hits to CAZymes by providing “competitors” for a best hit. We used the relative abundance of CAZymes in our analysis, calculated as the ratio of the number of reads annotated to a given CAZyme to the total number of reads to which any Pfam was annotated. Because the IMG annotation pipeline used family-level trusted cutoffs only for annotating Pfams in one Prospect Hill organic horizon-warmed sample, that sample was excluded from the functional analysis.

### Presence of differentially abundant genes.

All statistical analyses were completed in R (version 3.1.0 [http://www.r-project.org]). Plots were generated using ggplot2 version 1.0.1 (41). A negative binomial model (42, 43) was used to identify genes or taxa that were differentially abundant between warmed and control plot samples using MASS version 7.3-43, and then corrected *P* values from Wald tests were used to account for the false-discovery rate (44). A warming effect was assessed individually for each experiment as well as for all experiments together by pooling experiments and analyzing each horizon separately. Model fit was verified using a chi-square test. Due to the small number of replicates (3, 4) for within-experiment comparisons, warming effects were considered significant at a corrected alpha of 0.1 (45). The overall warming effect (all sites) was deemed significant at a corrected alpha of 0.05. The effect of warming treatment on the relative abundance of Pfam reads annotated as CAZymes was determined and plotted as 100 × [(relative abundance in heated − relative abundance in control)/(relative abundance in control)]. The taxonomic affiliations of carbohydrate-degrading genes were plotted similarly. Because there are either no blocks in the design of each experiment (Barre Woods and SWaN) or we did not sample warmed and control plots within the same experimental block (Prospect Hill), there are no natural warmed-control sample pairs in our sequencing design. Therefore, we used a bootstrapping approach (999 iterations) to generate estimates of the standard error of the ratio for plotting in our figures. Gene families indicative of warming treatment were additionally identified using random forest analysis with 1,000 trees, followed by feature selection using the Boruta algorithm (46) at a *P* value of <0.01. In contrast to other feature selection methods that look for the minimal predictive set, Boruta identifies all genes that assist with group prediction, including those that offer redundant information (46). The final reduced set of genes was evaluated for its ability to predict warming treatment of origin using a second random forest analysis. The analysis was not repeated by site due to inadequate replication. Overall changes in the phyla to which the carbohydrate-active enzymes were annotated were assessed using a permutational multivariate analysis of variance (adonis, vegan version 2.3-5 [https://cran.r-project.org/web/packages/vegan/index.html]) on Hellinger-transformed data.

### Isolate cultivation.

Dominant members of the bacterial communities at Prospect Hill (20 years of warming) were targeted for cultivation, with successful isolation of 537 dominant and rare organisms in 6 phyla from both warming treatment and control treatment soils (see Fig. S5 in the supplemental material). Soils from Prospect Hill were selected for cultivation because microbial communities are known to have shifted with warming at this site (4, 16, 22). Soils were collected on 22 October 2013, 28 April 2014, and 30 June 2014 when we were sampling the site for other purposes. On these instances, soil samples were collected with 1/2-in. tubular soil corers to a depth of 10 cm and split by eye into organic and mineral soils. The soil corer was washed with 70% ethanol between plots to minimize cross-contamination.

Various methods known to be effective for isolating the closest cultured relatives of dominant Acidobacteria, Actinobacteria, and Alphaproteobacteria identified in a previous study of these soils were used (19). Our overall approach was to maximize the total number and diversity of bacteria isolated from soil, with the intention of examining how warming changed the phylogeny of biopolymer-degrading traits. We used low-nutrient media and long incubation times (weeks to months), as these have been shown to increase up to a hundredfold the number and diversity of bacteria that can be cultured (47). Soil treatment methods prior to isolation on solid medium included (i) placing lignin-amended Bio-Sep beads into warmed and control plots for 3 months (4) and then transferring them to a minimal medium (48) with Kraft lignin as sole C source under anaerobic conditions for 6 weeks, (ii) diluting soil to extinction in a soil solution mimic (49) and growing the resultant dilutions under aerobic conditions at room temperature prior to plating on solid medium, (iii) drying soils at 120°C for 1 h with or without a 1-h 30°C phenol treatment designed to select for Gram-positive organisms, (iv) surface-sterilizing and then grinding plant roots in an extraction solution to select for endophytic bacteria (50), and (v) vigorously stirring soil in the presence of 2.24 mM dithiothreitol and 1 mM sodium pyrophosphate for 1 h prior to plating to enable the separation of bacteria from soil while minimizing oxidative stress (51). Media used included oatmeal agar (52), humic acid vitamin agar (53), lignin soybean flour vitamin agar (54), MM1 (55), 1% nutrient agar (56), modified carboxymethyl cellulose (CMC) (50), water agar plus yeast extract, and VL-55 with gellan gum, xylan from birchwood, carboxymethyl cellulose, pectin, xanthan gum, or readily oxidized carbon (51). Plates were incubated in the dark at 23 to 25°C in the laboratory at room temperature in an anaerobic Coy chamber filled with 5% H_2_, 5% CO_2_, 90% N_2_, or with switching between these conditions every 2 to 3 days to mimic the reduced oxygen conditions characteristic of soil. We also prepared roll tubes with a 1 to 2% oxygen headspace (57) and incubated them at 18°C. In all instances, no-soil negative controls were prepared in order to rule out the possibility of cross-contamination between warmed and control plot soils. Plates and tubes were incubated from 1 week to 6 months before colonies were picked. No obligate anaerobes were identified. All bacteria were streaked to isolation under aerobic laboratory conditions and then cryopreserved in 15% glycerol at −80°C. Freezer stocks were identified and verified as pure by sequencing of 16S rRNA gene PCR products using the primers 27F (5′-AGAGTTTGATCMTGGCTCAG-3′) and 1492R (5′-TACGGYTACCTTGTTACGACTT-3′) (58). Sequences were trimmed to include only an average quality score of >60 and examined for contamination in 4Peaks (version 1.7.2 [Nucleobytes]) before being assigned taxonomic categories using EzTaxon (59). Closely related isolates were identified by clustering 16S rRNA sequences at 99% identity using cd-hit (60), and BLASTn (61) was subsequently used to map metagenome 16S rRNA reads to these clusters.

A phylogenetic tree (Fig. 4) of isolate sequences was built with RAxML version 7.7.2 (62) (100 bootstraps, GTRGAMMA model of nucleotide substitution) using sequences aligned using the bacterial model in RDP (33). We only included those 295 bacteria that both demonstrated growth in at least two of the three substrate use assays and for which we had viable freezer stocks verified as pure by sequencing of the 16S rRNA gene in our tree. Methanocaldococcus jannaschii DSM 2661 was included as the outgroup. The 16S rRNA sequences of the following strains were extracted from NCBI's 16S prokaryotic rRNA database (63) and included in the isolate alignment to assist with tree building: Terriglobus roseus KBS 63, Burkholderia soli GP25-8, Nevskia terrae KIS13-15, Bradyrhizobium lablabi CCBAU 23086, Flavobacterium soli KCTC 12542, Opitutus terrae PB90-1, Bacillus subtilis NRS 744, Isosphaera palida ATCC 43644, and Mycobacterium smegmatis 987-M10. The phylogenetic tree was drawn and annotated using iTOL (64). Isolation information for bacteria used in this analysis can be found in Data Set S2 in the supplemental material.

### Physiological characterization of isolates.

Isolates were characterized for the ability to depolymerize three polysaccharides if their cryopreserved cultures had been validated as pure and growth was observed on the assay medium used. Our objective was to evaluate how long-term warming has affected the ability to degrade polysaccharides in phylogenetically diverse organisms isolated from soil, with the expectation that a greater fraction of bacterial genotypes from heated plots would be able to degrade biopolymers. All incubations and assays were completed aerobically at room temperature (23 to 25°C), as these are the conditions under which all isolates had been maintained following isolation. With a few exceptions for very slow-growing isolates, isolates were streaked from freezer stocks onto 10% tryptic soy agar (TSA) 6 days prior to characterization. Three distinct colonies were selected, and each was inoculated into 10 ml of 10% tryptic soy broth (TSB) and allowed to grow for 24 h with gentle shaking (120 to 150 rpm). Ten microliters of this culture was inoculated onto solidified medium with 0.1% chitin, carboxymethyl cellulose (TCI C0045), or xylan (catalog no. X4252; Sigma) for evidence of depolymerization, where depolymerization is seen as zones of transparency around growth on otherwise-opaque medium for chitin, and as zones of yellow on otherwise-purple-brown medium following staining with Gram's iodine for xylan and cellulose (65). Chitin from crab shells (catalog no. C-7170; Sigma) was colloidized for inclusion in medium by soaking 15 g in 112.5 ml of 12 M hydrochloric acid with periodic stirring for 1 to 2 h, and then precipitating it in 3.375 liters of ice-cold distilled water overnight, filtering the pellet, and rinsing it with ice cold phosphate-buffered saline (pH 7.5) until the eluent reached circumneutral pH. We used MM medium (66) as the base for plate-based assessment of zones of clearance. Chitin degradation was examined after 11 days of growth, while xylan and cellulose degradation was assessed after 4 days. The bioinformatic tool consenTRAIT (5) was used to calculate the mean phylogenetic trait depth for groups where at least 90% of the members are able to use a substrate (τ_D_). Significant differences in isolate potential for biopolymer utilization were assessed using a phylogenetic logistic regression method implemented in the phylolm package in R (version 2.4 [https://cran.r-project.org/web/packages/phylolm/index.html]), using 1,000 bootstraps (67).

### Accession number(s).

Metagenomes and all annotations are available in IMG (https://img.jgi.doe.gov/) under taxon object identification (ID) numbers 3300001606 to 3300001636, 300001638 to 3300001650, 3300001652, 3300001653, 3300001658, and 3300002954, and in NCBI's Sequence Read Archive under accession numbers SRX2013848, SRX2014317 to SRX2014349, SRX2014378 to SRX2014381, SRX2014384 to SRX2014388, SRX2014390, SRX2014391, SRX2014394, SRX2014397, and SRX2014398.

## RESULTS

### Diversity of carbohydrate-degrading genes.

The metagenomes were dominated by bacteria, which accounted for an average of 97.8% ± 0.06% of the protein-coding reads and 91.6% ± 0.5% of the rRNA reads across all samples. Independent of the annotation method, Actinobacteria, Acidobacteria, and Proteobacteria accounted for the majority of annotated metagenome reads (see Fig. S1 in the supplemental material). In contrast, eukaryotes accounted for 1.53% ± 0.07% of protein-coding reads. Carbohydrate-active enzyme genes were identified by mapping Pfam IDs (37) to the CAZy database (40) and included glycoside hydrolases, glycosyltransferases, polysaccharide lyases, and carbohydrate binding motifs (see Fig. S2 in the supplemental material). As expected based on annotated genomes, these genes accounted for 1.59 to 2.08% of the protein-coding genes present in the metagenomes (40), with similar relative abundances at Prospect Hill and SWaN and a lower relative abundance at Barre Woods (see Fig. S2). There are 162 carbohydrate-active enzymes for which a Pfam-to-CAZy match is possible, and all were identified in our metagenomes. Of these, glycoside hydrolases associated with alpha-glucosidase linkages (GH13) and glycosyl-transferases (GT4) were the most abundant (see Fig. S3 in the supplemental material). The diversity of CAZymes was generally unaffected by warming treatment, with unchanged Shannon index [*H* = 4.04 versus *H* = 4.06, *t*(4.91) = 2.31, *P* = 0.070] and a small but significant decrease in Pielou's evenness with warming [heated *J* = 0.80 versus control *J* = 0.81, *t*(4.73) = 2.82, *P =* 0.039] in the organic horizon at Prospect Hill. Based on a permutational analysis of variance (ANOVA), the taxonomic composition of individual CAZymes was driven first by soil horizon (*R*^2^ = 0.37, *P* = 0.001), then by site (*R*^2^ = 0.055, *P* = 0.039), and finally by warming treatment (*R*^2^ = 0.034, *P* = 0.037).

### Effects of warming on abundance and diversity of carbohydrate-degrading potential.

Metagenomic sequencing of soils from the three warming studies revealed that chronic warming significantly altered the repertoire of carbohydrate-degrading genes in an experiment-specific manner. The number of carbohydrate-degrading genes as a fraction of all annotated reads was significantly increased by warming, but only for the shortest (5-year warming) and longest (20-year warming) duration experiments, which are colocated within the experimental site away from the site warmed for 8 years (see Fig. S2 in the supplemental material). At the 20-year site, warming increased carbohydrate-active genes by 2.90% in the mineral horizon [*z*(7) = 31.8, *P =* 0.0015]. In the organic horizon, warming decreased the abundance of carbohydrate-active genes by 4.57% [*z*(7) = −4.81, *P* < 0.001] and decreased the abundance of glycoside hydrolases by 6.8% (*P* = 0.021). At the 5-year site, warming increased (4.45%) the fraction of genes annotated as carbohydrate active in the organic horizon [*z*(7) = 2.09, *P =* 0.037].

**FIG 1:**
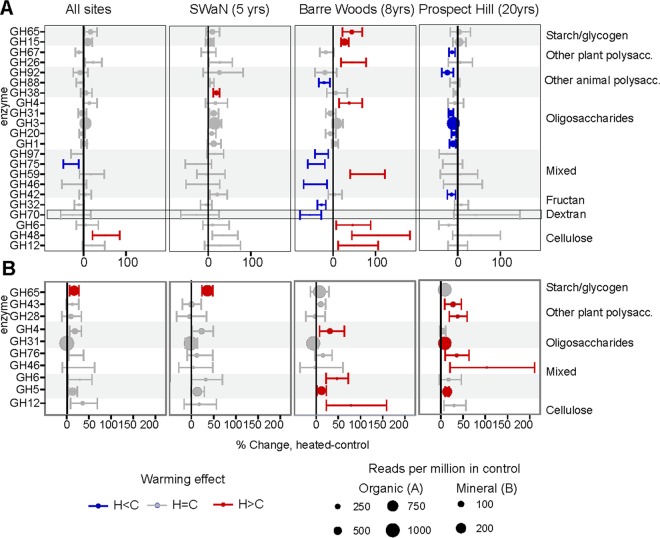
Effect of chronic warming on the relative abundance of polysaccharide (polysacc.)-degrading genes in organic (A) and mineral (B) soil. Points denote mean percent difference in relative abundance of CAZymes between heated (H) and control (C) plots as a fraction of annotated reads, and are colored where regression coefficients of a negative binomial model regression differed between warmed and control plots (Benjamini-Hochberg corrected Wald test, *P* < 0.1 for individual sites, *P* < 0.05 for all sites together). Symbol size is proportionate to genome-standardized abundance in the control plots. Panels are separated into all sites analyzed jointly to look for an overall warming effect (“all sites”) or separately by site, in increasing order of experiment age.

When all experiments were taken together, three genes showed a consistent response to warming (Fig. 1). In the organic horizon, warming was associated with decreased chitosanase genes (GH75) and with increased cellobiohydrolase genes (GH48). In the mineral horizon, warming was associated with increased alpha-glucosidase genes (GH65), a family that mostly contains sugar phosphorylases. Random forest analysis followed by Boruta feature selection identified an additional five gene families predictive of warming treatment in the organic horizon and 11 in the mineral soil (Table 2). The enzyme families identified by this method are able to degrade a variety of compound classes, including starch, chitin, and cellulose.

**TABLE 2:** CAZy gene families identified as indicative of warmed or control plot metagenomes

Warmed or control	Mineral		Organic horizon	
	Indicator gene families	Classification error rate (%)^*a*^	Indicator gene families	Classification error rate (%)
Warmed	GH26, GH4, GH46, GH65, GH66, GH76, GT4, GH48, GH52, CBM2, GT1, GT4	25.0	GH48, GH85	36.4
Control	GT35, GT5	16.7	GH75, GH100, CBM5, GT13, GH98	16.7

a Error refers to fraction of samples incorrectly assigned to warming treatment when just using the subset of genes identified as indicators by the Boruta algorithm at *P* value of <0.01. Example: if the relative abundance of each of GH75, GH100, CBM5, GT13, and GH98 was to be taken from a control plot mineral soil sample and used to try and determine whether that sample came from a warmed or control plot, 16.7% of the time, the sample would be incorrectly assigned as coming from a heated plot.

To determine whether the abundance of specific carbohydrate-active enzyme families was changed by warming, we examined data from each site individually. Warming treatment only increased two gene families at the 5-year site (SWaN). At the 8-year site (Barre Woods), almost as many gene families increased as decreased with warming in the organic horizon (Fig. 1). However, genes associated with cellulose degradation consistently increased with warming in both mineral and organic horizons. At the 20-year site (Prospect Hill), individual carbohydrate-degrading gene families significantly affected by warming increased in abundance in the mineral horizon and decreased in abundance in the organic horizon. After 2 decades of warming in these soils, gene families responsible for oligosaccharide degradation were consistently negatively affected by warming in the organic horizon.

**FIG 2:**
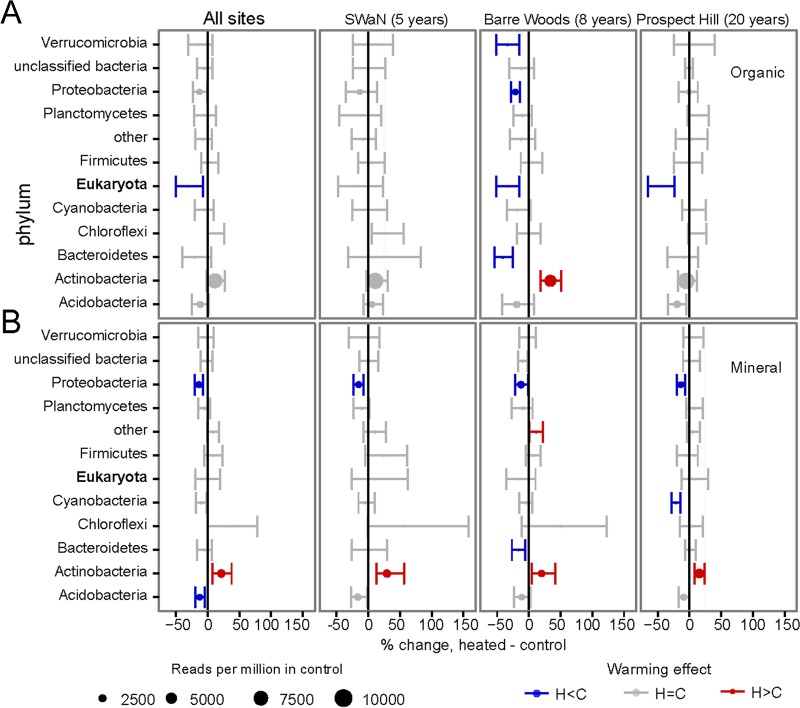
Effects of experimental warming on the fraction of annotated reads assigned to dominant phyla in organic (A) and mineral (B) soil. Circles are plotted as the percent difference between warmed and control plot values, with the size proportionate to the number of polysaccharide-associated reads in the metagenome assigned to the phylum. Other parameters are as per Fig. 1.

Actinobacteria, Acidobacteria, and Proteobacteria dominated the carbohydrate-degrading gene pool and shifted in dominance with warming (Fig. 2). In the mineral horizon in all experiments, warming treatment increased the fraction of reads annotated to Actinobacteria carbohydrate-degrading genes while decreasing the fraction annotated to Proteobacteria (Fig. 2). Only at Barre Woods, the experiment warmed for 8 years, did warming treatment have a similar effect in the organic horizon, increasing Actinobacteria and decreasing proteobacterial carbohydrate-degrading genes (Fig. 2). Warming also decreased the relative abundance of carbohydrate-degrading genes annotated to Eukaryotes in the organic horizon at the two longer-running experiments (Fig. 2). This was consistent with a 22.4% decrease in the eukaryotic-to-prokaryotic rRNA gene ratio with warming for all experiments [beta regression, *z*(23) = 2.17, *P =* 0.030].

**FIG 3:**
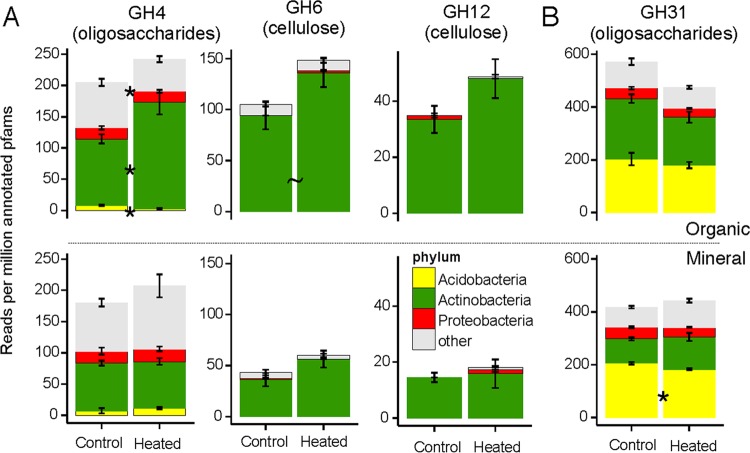
Taxonomic distribution of polysaccharide-degrading genes for which the overall abundance was significantly affected by warming in both the organic horizon (top row) and mineral soil (bottom row); see Fig. 1. Differences in fractions of Pfam reads assigned to a given taxon and function were analyzed using a *t* test with Benjamini-Hochberg correction. (A) Barre Woods. (B) Prospect Hill. No genes were affected by warming treatment in both horizons at SWaN. “Other” includes all reads identifiable to at least the domain level. ∼, *P* < 0.1; *, *P* < 0.05.

Some polysaccharide-degrading genes significantly affected by warming treatment in both soil horizons had altered phylogenetic distributions (Fig. 3). A substantial fraction of reads for the three enzymes that increased with warming in both soil horizons at Barre Woods were annotated to Actinobacteria. However, the number of reads annotated to Actinobacteria significantly increased with warming only for GH4 in the organic horizon. Although warming increased the overall abundance of GH31 in the mineral horizon at Prospect Hill, the number of reads annotated to Acidobacteria decreased with warming treatment. In contrast, although warming treatment decreased the overall abundance of GH31 by 16.9% in the mineral horizon, the number of reads for this gene annotated to individual phyla was unaffected.

### Carbohydrate-degrading capacity of isolated bacteria exposed to 20 years of warming.

We characterized the capacity of 295 bacterial isolates in 6 phyla (10 classes, 14 orders, and 34 families) from warmed and control treatments at Prospect Hill (20-year experiment) to degrade the carbohydrates carboxymethyl cellulose (CMC), xylan, and chitin. These organisms were cultivated as representatives of the diversity of organisms found in our soils, not for their ability to degrade these polysaccharides. Including those organisms, we did not have satisfactory sequence or substrate utilization data to incorporate into our phylogenetic tree; our isolate collection contains representatives of all dominant phyla found in the metagenome but is enriched in Firmicutes and Betaproteobacteria and depleted in Acidobacteria (see Fig. S4 in the supplemental material). Approximately 70% of the isolates showed at least 99% identity over the 16S rRNA gene with at least one other isolate, resulting in 103 clusters of closely related isolates. We found a significant phylogenetic signal-to-substrate depolymerization (greater-than-expected phylogenetic clustering of identical trait states where the phylogenetic correlation parameter *a* > −4; *a* = 25.6, 31.8, and 10.3 [67] and Fritz and Purvis's phylogenetic signal metric −*D* + 1 > 0; −*D* + 1 = 0.251, 0.393, and 0.500 [68, 69] for CMC, xylan, and chitin, respectively). However, these traits were only conserved to a mean 16S rRNA depth (τ_D_) (5) of 0.01 or less in all cases, indicating that even “conspecific” organisms sharing 99% 16S rRNA identity do not always share the ability to degrade a given polysaccharide in our assay. This is typified by the observation that isolates clustered at 99% 16S rRNA identity showed divergent substrate utilization patterns on many occasions, even for organisms isolated from the same warming treatment (see Table S2 in the supplemental material).

**FIG 4:**
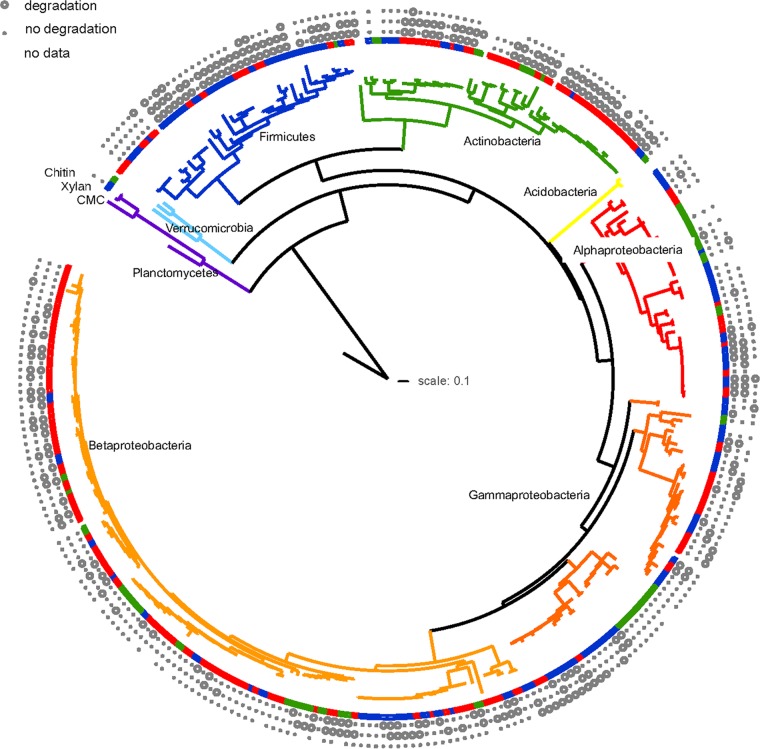
Phylogenetic tree of bacterial isolates collected from warmed and control plots or immediately adjacent to experimental plots at Prospect Hill. Branches are colored according to phylum or class (for Proteobacteria). Inner ring of colors denotes whether isolate came from warmed (red) or control (blue) plots or outside the plots (green), while outer rings denote whether the isolate was able to degrade the polymer in a 4-day (CMC and xylan) or 11-day (chitin) assay on solid medium, as shown in the key. Breaks in color ring denote type strains inserted for orientation. Archaea were removed from the tree after building. Positions with a color in the color ring but no data for any substrate failed to grow in the plate-based assay.

Despite divergent warming responses of conspecifics, a phylogenetic logistic regression showed that exposure to long-term warming significantly increased the probability of CMC and xylan depolymerization by isolates (logistic regression coefficient *b*_0_ = 0.707, *P =* 0.0040 and *b*_0_ = 0.641, *P =* 0.0073) (Fig. 4). Chitin depolymerization was not significantly affected by warming (*b*_0_ = 0.453, *P =* 0.13).

## DISCUSSION

We expected that warming treatment would increase the carbohydrate-degrading potential of the microbial community in the 20-year experiment. We found this to be the case in the mineral soil only; warming significantly decreased the fraction of genes annotated as being capable of degrading carbohydrates in the organic horizon. However, at the 5-year experiment, there was an increase in the relative abundance of biopolymer-degrading genes in the organic horizon. These results suggest that warming may have enriched for a carbohydrate-degrading gene pool in the organic horizon in the short term but eventually led to a depletion of these genes as the organic matter was increasingly degraded and/or translocated to the mineral horizon. Since organic horizon communities are enriched in carbohydrate-active enzymes compared to mineral soil communities (15), organic matter that is incompletely degraded in the organic horizon may enter the mineral horizon as particulate organic matter, promoting an increased abundance of carbohydrate-degrading genes in the mineral horizon. Warming treatment may increase the translocation of partially degraded organic matter into the mineral horizon by increased invertebrate activity in summer (70) or increased soil surface freeze-thaw outside the growing season (71). Such increased mixing of the organic and mineral horizons is consistent with our previous observation that bacterial communities in warmed organic and mineral soils are more similar to one another than communities in control organic and mineral soils are in these soil samples (16). Therefore, we posit that warming may cause an immediate loss of soil organic matter (SOM) primarily from physically unprotected pools in the organic horizon, but over time, the mineral horizon plays a more important role in positive feedback to climate. However, quantification of particulate organic matter in the upper mineral soil is necessary to validate the hypothesis of increased translocation from the organic horizon. Furthermore, additional annual and seasonal time points must be sampled to verify whether the observed changes in microbial community structure and function are consistent over time. Finally, while sites were selected to be as similar as possible for the warming experiments, edaphic differences between sites (Table 1) cannot be ignored as possible explanations for the differences in the horizon specificity of the warming response.

Our results also indicate that bacterial biopolymer degradation may be increasingly important to soil organic C cycling in a warming world. Fungi tend to dominate the microbial biomass and decomposition of litter in temperate forests (72) but generally form just a small fraction of metagenome reads (12). We observed a small and declining eukaryote contribution to both the polysaccharide-degrading gene pool and the overall gene pool in the organic horizon with warming in the present study. Because of the poor representation of environmentally relevant eukaryotes in genomic databases (73), it is possible that the decrease in eukaryotes is an artifact of our annotation method. However, we believe this is unlikely, because both quantitative PCR (qPCR) data from the present soil samples (16) and phospholipid fatty acid (PLFA) analysis (22) from the 20-year site showed a trend toward reduced fungal abundance and increased or unaffected bacterial abundance with warming. A strong extraction or annotation bias against fungi is also unlikely, as RNA coextracted with and analyzed in the same manner as the metagenomes used in the present study contained an average of almost 25% fungi. The fungus-to-bacterium biomass ratio has also been observed to decrease with warming in many other studies (74), with key exceptions occurring where mycorrhizal fungi have accompanied an expansion of host plants (75, 76). One possible mechanism for this shift in the microbial community responsible for carbohydrate degradation is through reduced soil carbon content; fungal biomass tends to increase with organic matter content and C/N ratio (77), and soil C tends to decrease with warming (78). An additional plausible mechanism for reduced fungal relative abundance under warming at the 8-year site only is a reduction in plant host tissue available for colonization; fine-root biomass was reduced by warming in this experiment (18). Distinguishing between these two hypotheses is not possible with the data used in the present analysis but could be important for understanding how plants and microbes may interact to drive ecosystem-level feedback to climate warming.

Warming increased the fraction of carbohydrate-degrading genes annotated to Actinobacteria in the mineral horizon at all sites and in the organic horizon at Barre Woods. This is consistent with a global trend toward increased relative abundance of this phylum under warming (74), including in metagenomes of a decade-long prairie warming experiment (12). Although members of the Actinobacteria show considerable variability in their ability to degrade carbohydrates (79), the genomes of Actinobacteria in our metagenomes were on average enriched in glycoside hydrolases responsible for cellulose, starch, and xylan degradation compared to other phyla based on a CAZyme-to-RNA polymerase subunit B standardization (see Fig. S5 in the supplemental material). A substantial fraction of individual carbohydrate-active genes belonged to Actinobacteria and were significantly increased by warming, suggesting an increasing role for Actinobacteria in the degradation of carbohydrates with warming. However, alternative explanations for the increased relative abundance of Actinobacteria cannot be excluded, such as an ability to better tolerate altered abiotic conditions related to warming, such as drier soils (80), compared to other taxa. Indeed, soil moisture was generally lower in heated plot soils (Table 1). These results also do not preclude the importance of other bacterial taxa in the degradation of soil carbohydrates; soil Actinobacteria may be overrepresented in genomic databases compared to other phyla due to their biotechnological relevance (81).

In line with previous studies (79), we found that carbohydrate degradation was phylogenetically conserved. Due to cultivation bias (82), phylogenetic correction is required to conclude increased probability of carbohydrate degradation ability (83), although many previous papers comparing the physiological capacity of bacteria isolated from different environments or under different conditions in the laboratory have failed to consider the potential need to correct for phylogenetic autocorrelation in their analysis (84–86). With this analysis of isolate physiology, we were able to establish that there is a trend toward increasing capacity of bacteria for degradation of cellulose and xylan with warming. The fact that an increased probability of degrading chitin was not observed in isolates from heated plots may be explained by reduced absolute (16, 22) and relative (this study) eukaryote abundance, since fungal cell walls and arthropod exoskeletons are the primary sources of chitin in soil (87). However, we caution that since the collection of bacteria isolated and characterized for biopolymer degradation in the laboratory is not entirely representative of the diversity and abundance of organisms in our study sites, applying this finding directly back to soils would be flawed. Nonetheless, the cultivation and characterization approach taken in the present study affords the unique opportunity to observe the functions demonstrated by individual bacteria, where metagenomics only enable examination of the potential function of the soil metaorganism. Furthermore, cultivation of conspecific organisms from dissimilar environments may eventually provide insight into the physiological basis of microevolutionary trends shown by bacteria in response to climate change.

While metagenomics and isolate cultivation can provide a reasonable evaluation of the range of phenotypes organisms can take on, *in situ* function is contingent upon numerous factors not readily captured in the present study. These include spatial and temporal heterogeneity in substrate availability and community structure (88), seasonal differences in transcriptional response (89), expression of isoenzymes and/or uptake transporters with different *K_m_*/*V*_max_ tradeoffs (2), gene-gene interactions (90), and interactions between substrate quality, quantity, and microbial physiology (24). Furthermore, metagenomics is liable to access the genomes of both active and inactive, living and dead cells (91). Intensive physiological assessment of dominant bacteria coupled to field-based studies of the *in situ* transcriptional or proteomic profiles of these organisms holds promise for closing a number of these gaps.

Soil organic matter decomposition is directly promoted by elevated temperatures in the short term (3, 78), and our study suggests that chronic climate warming may also indirectly boost decomposition by favoring those microbes with the ability to degrade common litter polymers. Although the distinct start times and geographic locations of the three warming experiments mean they form an imperfect chronosequence, our results further indicate that the effect of warming on the potential for carbohydrate degradation may vary with duration of warming and depth of soil. Soil respiration at the 20-year experiment has shown a triphasic respiration response (23, 92); our observation that the microbial community was enriched in genes for carbohydrate degradation in the organic horizon of the 5-year experiment and mineral soil of the 20-year experiment suggests that the secondary burst of respiration may have originated from the mineral soil. In the mineral soil at the 20-year site, as at all experiments in the present study, Actinobacteria increased in relative abundance, indicating that Actinobacteria may have been important for sustained carbon loss from these soils at the time of soil collection. Nonetheless, since we sequenced metagenomes from just a single time point, further assessment of possible interannual and seasonal variability in the drivers of microbial response to warming is necessary to corroborate our results.

An improved understanding of the effects of climate change on soil decomposers is increasingly recognized as vital for predicting soil carbon content. Substantial progress has been made in improving our knowledge of soil-level (20) and ecosystem-level (18, 78) responses to warming, but it is difficult to extend this result to new sites and conditions without a mechanistic understanding of how the key microbial players are responding to these changes (93). In contrast to much of the previous work at our experimental sites, which has focused on how carbon stocks and flows are affected by warming (17–20), the present study proposes a possible mechanism by which warming-induced changes in soil carbon stocks and microbial communities may produce feedback to influence one another. As such, our results build on other studies (10–12) and show that an enhanced capacity to degrade carbohydrates may be part of a general microbial response to chronic warming that is shared across ecosystems.

## Supplementary Material

Supplemental material
