# Supplementary material for: Long-Term Warming Alters Carbohydrate Degradation Potential in Temperate Forest Soils
Source: Appl Environ Microbiol. 2016 Oct 27;82(22):6518–30. doi: 10.1128/AEM.02012-16 (PMC5086546; doi:10.1128/AEM.02012-16)
Supplement: Supplemental material [file supp_82_22_6518__index.html]

Supplemental material 

# Long-Term Warming Alters Carbohydrate Degradation Potential in Temperate Forest Soils

## Supplemental material

- Supplemental file 1 -

  Summary of sequencing and annotation statistics of metagenomes (Table S1), substrate utilization capacity of clusters of isolates showing >99% identity over the length of the 16S ribosomal RNA gene (Table S2), phylum-level distribution of metagenome reads by annotation method, warming treatment, and soil depth (Fig. S1), overall abundance of CAZymes in metagenomes (Fig. S2), relative abundance of carbohydrate-active enzymes present at >0.01% relative abundance in the metagenomes (Fig. S3), relative abundances of taxa in the isolate collection and metagenome (Fig. S4), and genome-standardized abundance of CAZymes (Fig. S5).

  PDF, 431K
- Supplemental file 2 -

  Summary of metagenomic sequencing (Data Set S1).

  XLSX, 237K
- Supplemental file 3 -

  Isolation information for bacteria used in this analysis (Data Set S2).

  XLS, 54K
