# Supplementary material for: Long-Term Warming Alters Carbohydrate Degradation Potential in Temperate Forest Soils
Source: Appl Environ Microbiol. 2016 Oct 27;82(22):6518–30. doi: 10.1128/AEM.02012-16 (PMC5086546; doi:10.1128/AEM.02012-16)
Supplement: Supplemental material [file AEM.02012-16_zam999117506so1.pdf]

	Mean	SEM	Max	Min	units
Metagenome Size	1,387,259,102	6,3331,106	2,474,318,996	796,642,853	bases/metagenome
GC content	61.6	0.20	64.4	59.9	percent/metagenome
Library insert size	270	NA	NA	NA	basepairs/insert
Scaffolds	7,012,272	285,726	11,445,986	4,104,504	number/metagenome
Predicted protein-coding genes	7,106,077	289,319	11,162,785	4,158,601	proteins/metagenome
Percentage of reads with predicted products	32.3	0.30	37.1	24.5	percent/metagenome
Relative abundance of RNA	0.24	0.003	0.20	0.28	percent/metagenome
Relative abundance of rRNA	0.078	0.0004	0.024	0.1179785	percent/metagenome
Fraction of RNA genes that are rRNA	0.32	0.001	0.44	0.11	rRNA genes/RNA gene metagenome
Reads per sample	27,059,049	1,133,965	60,685,304	17,631,916	QC-checked sequences/metagenome
QC-filtered merged paired end reads per sample	7,983,732	2,216,965	11,968,988	4,628,651	sequences/metagenome
Bacterial fraction of protein-coding genes	99.0	0.047	99.4	97.8	percent annotatable/metagenome
Eukaryotic fraction of protein-coding genes	0.71	0.051	1.95	0.38	percent annotatable/metagenome
Archaeal fraction of protein-coding genes	0.27	0.014	0.532	0.11	percent annotatable/metagenome
Viral fraction of protein-coding genes	0.012	0.0004	0.021	0.008	percent annotatable/metagenome

Table S1 : Summary of sequencing and annotation statistics of metagenomes

Cluster Number	Warming treatment	phylum	class	CMC	Xylan	Chitin	isolate count
0	control	Proteobacteria	Gammaproteobacteria	100	0	0	1
0	warm	Proteobacteria	Gammaproteobacteria	0	0	0	2
1	control	Proteobacteria	Gammaproteobacteria	100	100	0	1
1	warm	Proteobacteria	Gammaproteobacteria	100	100	0	1
5	control	Firmicutes	Bacilli	100	100	100	1
5	warm	Firmicutes	Bacilli	100	100	100	1
14	control	Proteobacteria	Betaproteobacteria	0	0	0	1
14	warm	Proteobacteria	Betaproteobacteria	50	50	0	5
15	control	Proteobacteria	Betaproteobacteria	0	0	0	2
15	warm	Proteobacteria	Betaproteobacteria	66	33	0	3
17	control	Proteobacteria	Betaproteobacteria	100	0	0	1
17	warm	Proteobacteria	Betaproteobacteria	100	0	0	1
19	control	Proteobacteria	Betaproteobacteria	0	50	0	2
19	warm	Proteobacteria	Betaproteobacteria	100	0	0	1
23	control	Proteobacteria	Betaproteobacteria	0	0	0	2
23	warm	Proteobacteria	Betaproteobacteria	0	0	0	4
25	control	Proteobacteria	Betaproteobacteria	0	0	0	2
25	warm	Proteobacteria	Betaproteobacteria	100	0	0	1
27	control	Actinobacteria	Actinobacteria	100	100	0	1
27	warm	Actinobacteria	Actinobacteria	100	100	0	3
33	control	Proteobacteria	Betaproteobacteria	37.5	25	0	8
33	warm	Proteobacteria	Betaproteobacteria	50	50	0	2
40	control	Proteobacteria	Gammaproteobacteria	25	25	100	4
40	warm	Proteobacteria	Gammaproteobacteria	0	0	100	1
53	control	Proteobacteria	Alphaproteobacteria	87.5	75	12.5	8
53	warm	Proteobacteria	Alphaproteobacteria	100	67	33	3
56	control	Actinobacteria	Actinobacteria	50	50	0	2
56	warm	Actinobacteria	Actinobacteria	85.7	100	14.3	7
98	control	Proteobacteria	Betaproteobacteria	0	0	0	1
98	warm	Proteobacteria	Betaproteobacteria	0	0	0	1
124	control	Proteobacteria	Betaproteobacteria	25	50	0	4
124	warm	Proteobacteria	Betaproteobacteria	25	50	0	4
125	control	Proteobacteria	Gammaproteobacteria	100	0	100	1
125	warm	Proteobacteria	Gammaproteobacteria	100	100	100	1
139	control	Proteobacteria	Betaproteobacteria	0	0	0	1
139	warm	Proteobacteria	Betaproteobacteria	0	100	0	1
183	control	Proteobacteria	Betaproteobacteria	100	100	0	1
183	warm	Proteobacteria	Betaproteobacteria	33	33	0	3
214	control	Actinobacteria	Actinobacteria	0	0	0	1
214	warm	Actinobacteria	Actinobacteria	0	0	100	1

Table S2 Substrate utilization capacity of clusters of isolates showing >99% identity over length of 16S ribosomal RNA gene. “warming” column denotes plot of origin, and values for CMC (cellulose), xylan, and chitin denote fraction of isolates within the cluster for heated or control plot isolates that are able to degrade the given substrate in a plate-based assay. Isolates indicates the number of isolates used to calculate the percentage. Only clusters with both heated and control plot members, and for which information for all three substrates was available are included in the table.

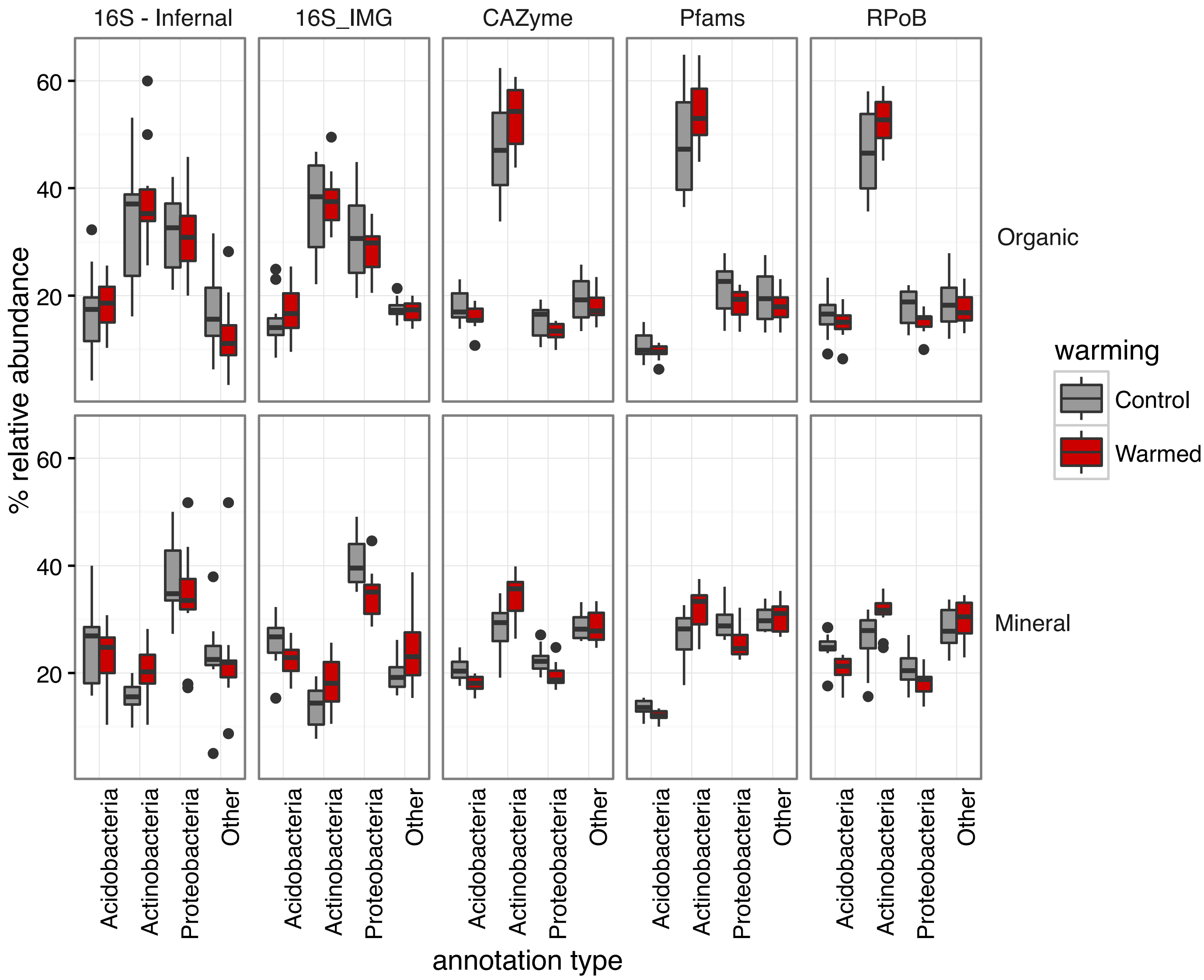

Figure S1: phylum-level distribution of metagenome reads by annotation method, warming treatment, and soil depth. CAZyme annotations are as described in the text, Pfam annotations are the full set of Pfams the CAZymes were matched to, and RPoB is just PF00562 in the Pfam annotations. 16S_IMG are the 16S reads identified by the IMG pipeline, which uses RNAmmer, Rfam, and Infernal (1), and annotated to phyla by clustering sequences with SILVA v119 at 90% identity using uclust. 16S_Infernal refers to unassembled metagenome reads identified using a covariance model in Infernal (2), and subsequently assigned taxonomy using RDP v2.11 (3) with a minimum phylum-level bootstrap of 0.5 and a domain-level bootstrap of 0.8.

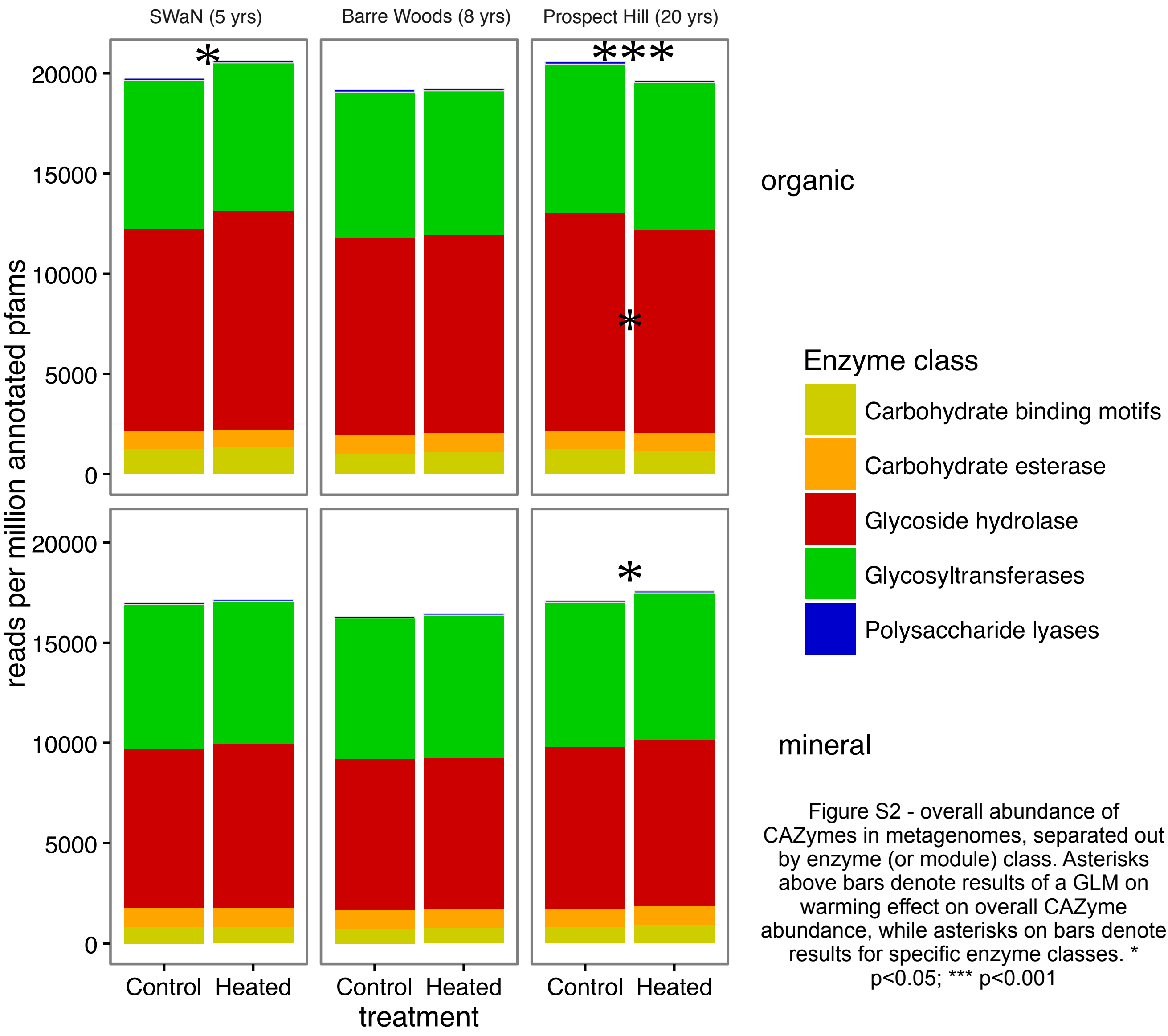

Figure S2 - overall abundance of CAZymes in metagenomes, separated out by enzyme (or module) class. Asterisks above bars denote results of a GLM on warming effect on overall CAZyme abundance, while asterisks on bars denote results for specific enzyme classes. * $p<0.05$; *** $p<0.001$

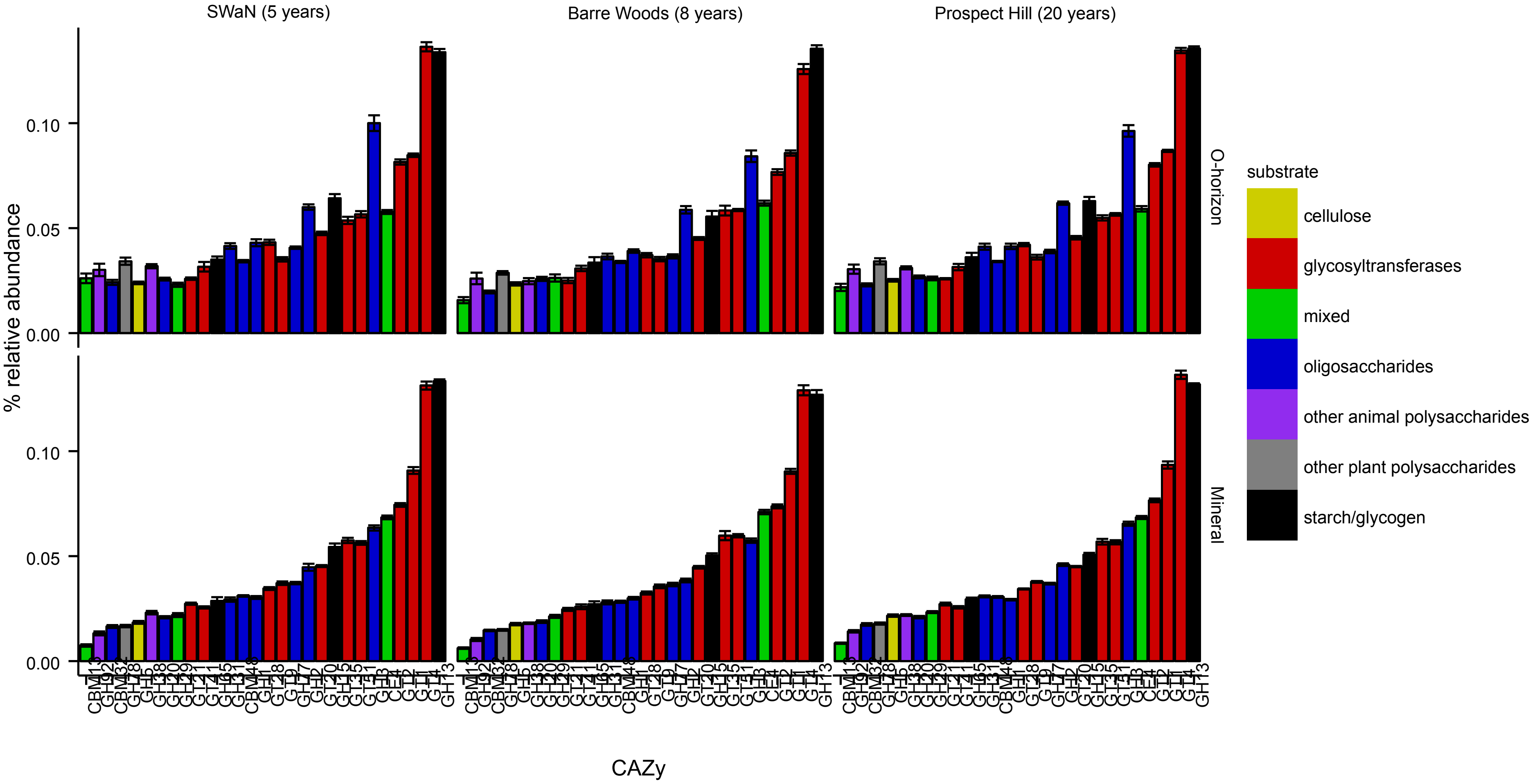

Figure S3 Relative abundance of carbohydrate-active enzymes present at >0.01% relative abundance in the metagenomes. Bars are coloured by substrate class. Genes present at significantly different abundance in heated and control plots can be found in figure 1 of the main text

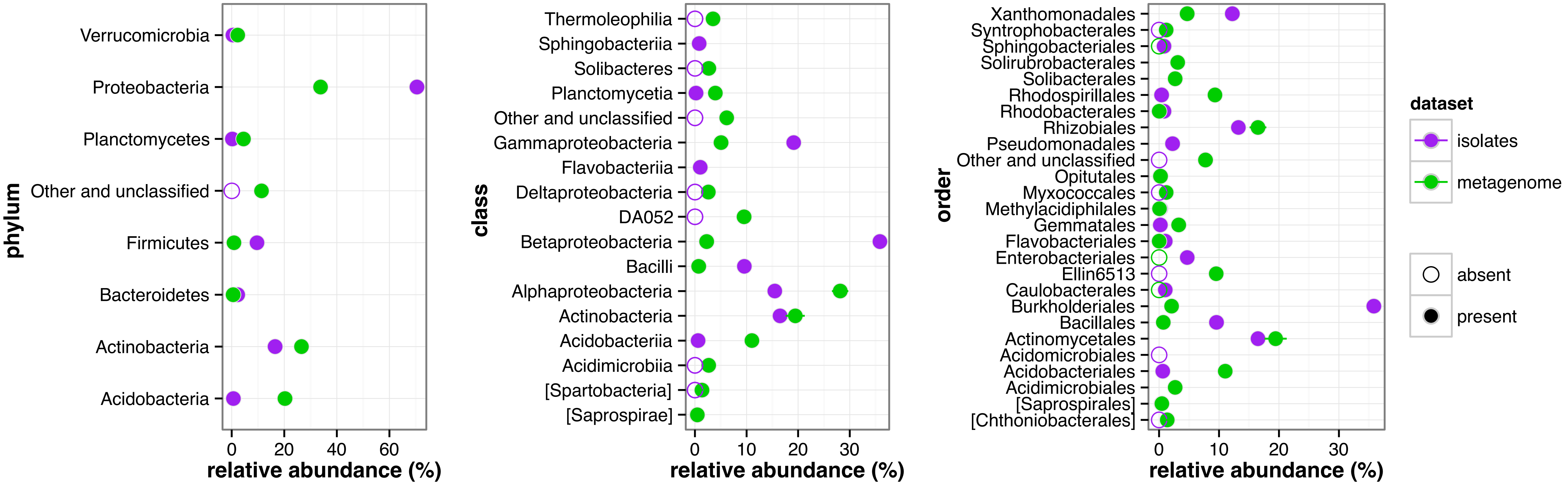

Figure S4: comparison of relative abundance of (A) phyla, (B) classes, and (C) orders in the isolate collection and metagenome, based on 16S rRNA genes. All isolate taxonomic groups are shown, but additional groups present only in the metagenome are shown only if they are present at greater than 1% relative abundance. Taxonomy was assigned using RDP. Open circles denote taxa not represented in a given dataset. Some taxa present in our isolate collection were absent from the metagenome reads, although they did appear in our more in-depth sampling of the phylogenetic diversity of these soil samples (4).

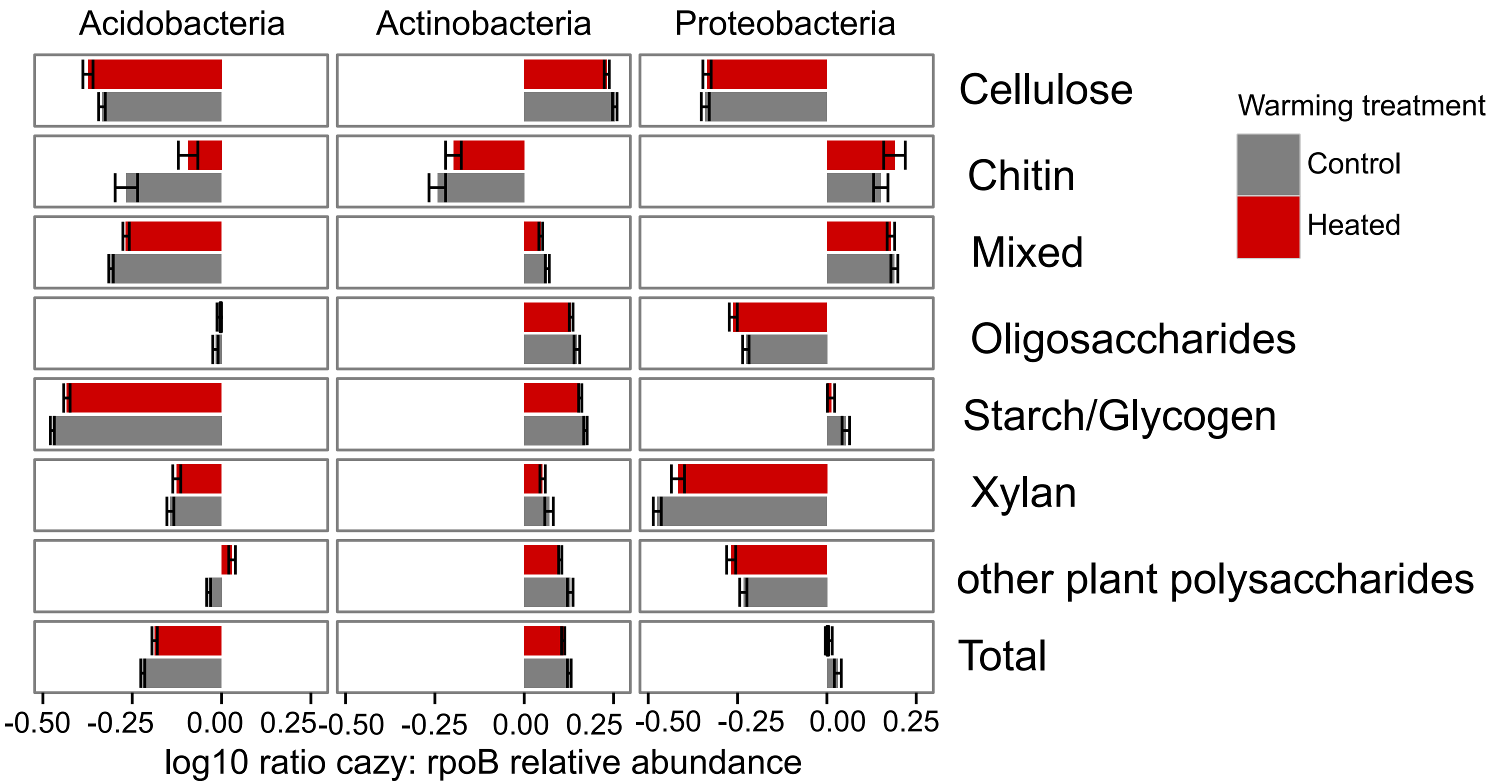

Figure S5: genome-standardized abundance of CAZymes. The number of RNA polymerase subunit B reads (a single copy bacterial gene) was summed for each phylum, and the fraction of RPoB reads annotated to each phylum of interest was determined. The same was done for CAZymes in each of the substrate categories of interest. Values plotted denote the log ratio of fraction of CAZyme genes annotated to a phylum to the fraction of RPoB genes, and therefore the values represent whether genes to degrade the substrate class of interest are present at greater (positive) or lower (negative) copies per genome than the “average” bacterium in the sample.

Supplementary references:

1. **Markowitz VM, Chen I-MA, Chu K, Szeto E, Palaniappan K, Grechkin Y, Ratner A, Jacob B, Pati A, Huntemann M, Liolios K, Pagani I, Anderson I, Mavromatis K, Ivanova NN, Kyrpides NC.** 2012. IMG/M: the integrated metagenome data management and comparative analysis system. Nucl Acids Res **40:**D123–D129

2. **Nawrocki EP, Eddy SR.** 2013. Infernal 1.1: 100-fold faster RNA homology searches. Bioinformatics **29:**2933–2935

3. **Wang Q, Garrity GM, Tiedje JM, Cole JR.** 2007. Naïve Bayesian Classifier for Rapid Assignment of rRNA Sequences into the New Bacterial Taxonomy. Appl Environ Microbiol **73**:5261–5267

4. **DeAngelis KM, Pold G, Topcuoglu BD, van Diepen LTA, Varney R, Blanchard J, Melillo J, Frey SD.** 2015. Long-term forest soil warming alters microbial communities in temperate forest soils. Terr Microbiol **6**:104
